# Implementing Real-Time Image Processing for Radish Disease Detection Using Hybrid Attention Mechanisms

**DOI:** 10.3390/plants13213001

**Published:** 2024-10-27

**Authors:** Mengxue Ji, Zizhe Zhou, Xinyue Wang, Weidong Tang, Yan Li, Yilin Wang, Chaoyu Zhou, Chunli Lv

**Affiliations:** 1 China Agricultural University, Beijing 100083, China; 2023308130430@cau.edu.cn (M.J.); zzz22@cau.edu.cn (Z.Z.); wangxy18@cau.edu.cn (X.W.); 2023308130229@cau.edu.cn (W.T.); liyan2023@cau.edu.cn (Y.L.); wangyilin2023@cau.edu.cn (Y.W.); zhouchaoyu2022@cau.edu.cn (C.Z.); 2 School of Computer and Cyberspace Security, Communication University of China, Beijing 100024, China

**Keywords:** hybrid attention mechanism, deep learning in agriculture, real-time disease detection, hybrid loss function, convolutional neural networks

## Abstract

This paper developed a radish disease detection system based on a hybrid attention mechanism, significantly enhancing the precision and real-time performance in identifying disease characteristics. By integrating spatial and channel attentions, this system demonstrated superior performance across numerous metrics, particularly achieving 93% precision and 91% accuracy in detecting radish virus disease, outperforming existing technologies. Additionally, the introduction of the hybrid attention mechanism proved its superiority in ablation experiments, showing higher performance compared to standard self-attention and the convolutional block attention module. The study also introduced a hybrid loss function that combines cross-entropy loss and Dice loss, effectively addressing the issue of class imbalance and further enhancing the detection capability for rare diseases. These experimental results not only validate the effectiveness of the proposed method, but also provide robust technical support for the rapid and accurate detection of radish diseases, demonstrating its vast potential in agricultural applications. Future research will continue to optimize the model structure and computational efficiency to accommodate a broader range of agricultural disease detection needs.

## 1. Introduction

In agricultural production, timely detection and the control of diseases are crucial for ensuring crop yield and quality [1,2,3]. Particularly in major agricultural production areas like Bayannur, where radish serve as a significant economic crop, disease management directly impacts farmers’ economic income and the sustainable development of regional agriculture [4,5,6]. Real-time and accurate disease detection technologies enable farmers to intervene effectively at the early stages of disease, thereby reducing the use of chemical pesticides, enhancing crop quality and safety [7,8], and further advancing the modernization and intelligentization of agriculture.

Traditional methods of radish disease detection primarily rely on visual observation by farmers or experts, a method that is not only inefficient, but also heavily dependent on individual experience [9]. With the expansion in agricultural scale and the increase in labor costs, these traditional methods are no longer adequate for large-scale agricultural production. Moreover, visual observation struggles to detect diseases at an early stage; once diseases spread, they can cause severe economic losses to farmers [10]. Thus, the development of an efficient, automated disease detection method has become urgent.

With the rapid advancement of computer vision and deep learning (DL) technologies [11,12,13], image-based methods for disease recognition have provided new solutions for the prevention and control of plant diseases [14]. These technologies enable the automatic identification and classification of different disease features by analyzing images of plant leaves, fruits, and other parts, thus achieving rapid and accurate disease detection. Vocaturo Eugenio et al. [15] explored the opportunities and challenges of AI-driven agriculture; Priyadharshini et al.’s study [16] utilized Convolutional Neural Networks (CNN), R-CNN, FAST R-CNN, and Faster R-CNN for detecting diseases in tomato leaves, demonstrating that the use of Faster R-CNN and VGG 16 achieved an accuracy exceeding 98%; Amit Kumar et al. [17] used Residual Networks (ResNet) to detect leaf diseases in crops, enhancing the main crops and economic benefits; Sachin Kumar et al. [18] employed pre-trained deep CNNs and ResNet on the cloud for classifying tomato leaf diseases. They conducted experiments with a dataset containing six different types of diseases and one healthy tomato leaf class. The results from ResNet-50 showed that for 50% training and 50% testing data, an accuracy of 96.35% was achieved, although actual tomato leaves were not collected for testing.

In response, Ankita Gangwar et al. [19] introduced a customized deep-learning-based convolutional vision transformer model. This model categorized tomato leaf images with simple and complex backgrounds into 13 classes, achieving an accuracy of 93.51%. It requires only 5.8 MB of storage space, which is 98.93%, 98.33%, and 92.64% lower than the state-of-the-art visual geometry group, vision transformers, and convolutional vision transformer models, respectively. Its training time is 44 min, which is 51.12%, 74.12%, and 57.7% lower than the aforementioned models. Therefore, it can be deployed on IoT-enabled devices, drones, or mobile devices to assist farmers in real-time monitoring of tomato crops. Vijaypal Singh Dhaka et al. [20] systematically demonstrated the role of integrating cutting-edge technologies, such as the Internet of Things (IoT) and DL in automating agricultural processes. They concluded that DL models like VGG-19, ResNet, DenseNet, AlexNet, and GoogleNet reported higher accuracy but suffered from poor storage efficiency. Nidhi Kundu et al. [21] proposed a deep-learning-based framework for dataset preprocessing, automatic disease detection, severity prediction, and crop loss estimation. It uses the K-Means clustering algorithm for extracting regions of interest. Subsequently, they employed the custom deep learning model “MaizeNet” for disease detection, severity prediction, and crop loss estimation. Experimental results showed their highest accuracy of 98.50%. Additionally, they also used Grad-CAM for feature visualization.

CNN-based models have significantly improved plant disease detection. However, specific models exhibit limitations, such as translation invariance, local sensitivity, and a lack of global image understanding [22]. Sana Parez et al. [23] proposed a fine-tuning technique based on Vision Transformers (ViTs), termed GreenViT, for detecting plant infections and diseases. Similar to word embeddings, they divided the input image into smaller blocks or patches and fed them sequentially to a ViT. Their method leverages the advantages of ViTs to overcome problems associated with CNN-based models. Experiments were conducted on widely used benchmark datasets to evaluate the performance of the proposed GreenViT. According to the results obtained, the proposed technique outperformed state-of-the-art CNN models in detecting plant diseases; Yang et al. [24] introduced a method incorporating a Dense High-Level Component Feature Pyramid Network (DHLC-FPN) into the DETR algorithm to detect rice diseases, with empirical results showing that the optimized structure for feature extraction significantly enhanced the model’s average detection precision and accuracy for small targets, achieving an average precision of 97.44% on the dataset; Utpal Barman et al. [25] proposed a smartphone-based solution considering deployment issues, using a ViT model to distinguish between healthy and unhealthy plants afflicted with diseases. The collected tomato leaf dataset was used to collectively train DL models based on Vision Transformer and Inception V3 to differentiate healthy from diseased plants. This work also proposed a smartphone-based application using a ViT-based model, which operates on a self-attention mechanism, producing better performance than Inception V3 during testing (90.99%).

Given the typical temperate continental climate of the Bayannur area, characterized by dryness, high winds, and ample sunshine, these conditions, while conducive to the growth of crops like radish, can also trigger certain specific diseases. Thus, to adapt to this variable natural environment, an intelligent disease detection system capable of real-time response and efficient image data processing is required. The real-time radish disease detection system proposed in this paper, based on a hybrid CNN–Transformer approach, is designed to meet this need, equipped with the capability for field deployment and ensuring stable and reliable disease detection under various environmental conditions. This study proposes a hybrid model combining a CNN and Transformer, aimed at enhancing the accuracy and real-time capability of disease image detection. The specific innovations include:

- Hybrid model architecture: By integrating the strengths of a CNN and Transformer, a new hybrid model architecture has been developed. CNNs are extensively used in image processing due to their excellent local feature extraction capabilities, particularly excelling in image recognition and classification tasks. Transformers, in visual tasks, can understand the global content of images through a self-attention mechanism, capturing complex and abstract image features that traditional CNNs might overlook. This model structure not only combines the advantages of both, but also, through innovative fusion strategies, enables the hybrid model to simultaneously focus on the details and overall layout of the image, enhancing the accuracy and reliability of disease recognition.
- Real-time processing capabilities: Considering the stringent demands for detection speed in agricultural application scenarios, the computational process of the model has been optimized. Through carefully designed network structures and algorithm improvements, it is ensured that rapid identification and classification of radish diseases are achieved without sacrificing detection accuracy. This capability is particularly crucial for field applications, helping farmers to detect and manage diseases in a timely manner and effectively prevent the spread of diseases.
- Hybrid attention mechanism: To further enhance the model’s performance, a novel hybrid attention mechanism has been designed. This mechanism combines the advantages of spatial and channel attention, dynamically adjusting the network’s focus on different spatial locations and feature channels, effectively enhancing the model’s representation of disease features. This not only improves the accuracy of disease detection, but also enhances the model’s efficiency in processing complex images.
- Loss function design: A hybrid loss function has been proposed to optimize the model’s performance in terms of classification accuracy and regression stability. This loss function considers both classification errors and localization errors through precise weight adjustments, enabling precise control over the model training process. This design not only helps the model achieve higher accuracy in disease detection, but also ensures balanced performance across various evaluation metrics.

## 2. Related Work

### 2.1. CNN Networks

The foundational construct of CNNs is the convolutional layer, whose core function is to extract features from the input image [26,27]. The convolution operation is represented by the following equation:

(1) $$\mathrm{output}\left(x,y\right)=\sum_{i=-a}^{a}\sum_{j=-b}^{b}\mathrm{input}\left(x-i,y-j\right)\cdot \mathrm{kernel}\left(i,j\right)$$

where (x,y) denotes the spatial location on the output feature map, kernel(i,j) represents the weights within the convolutional kernel, and *a* and *b* define the size of the kernel. This operation enables CNNs to capture local dependencies and spatial hierarchies within the image.

#### 2.1.1. AlexNet

Introduced by Krizhevsky et al. in 2012 [28], AlexNet achieved breakthrough results in that year’s ImageNet competition, credited to several key technological innovations and design choices. Firstly, AlexNet utilized a deep network structure with five convolutional layers and three fully connected layers, a depth significantly surpassing prior neural network models. Deeper networks can learn richer and more abstract features, but they also introduce increased training challenges [29,30]. To address the common issue of vanishing gradients in deep network training, AlexNet incorporated the ReLU (rectified linear unit) as its activation function. The mathematical expression for ReLU is given by:

(2) ReLU(x)=max(0,x)

This activation function, unlike traditional sigmoid and tanh functions, maintains a constant gradient in the positive range, preventing gradients from diminishing to near zero across many layers, thus effectively mitigating the vanishing gradient problem. In real-time radish disease detection systems, the use of ReLU not only accelerates the training speed of neural networks, but also enhances the stability and responsiveness of the model when processing actual agricultural image data. Additionally, AlexNet incorporated Local Response Normalization (LRN) within its architecture, a technique that enhances the model’s response to high-frequency features in images, further improving image classification accuracy [20]. Although subsequent studies have suggested that the effects of LRN might not be as significant as anticipated, it played a role in enhancing network performance at the time.

#### 2.1.2. ResNet

In the field of DL, as network depth increases, so does the model’s expressive power, but this also introduces challenges in training, especially issues like gradient vanishing or explosion. The introduction of ResNet marked a significant advancement in deep network design [31]. The core idea of ResNet is the implementation of a residual learning mechanism, which greatly facilitated the training efficiency and accuracy of deep networks, profoundly influencing subsequent network designs [32]. The fundamental concept of ResNet is that if the learning objective is an identity mapping, it may be easier for the network to fit this identity mapping directly rather than learning the residual (i.e., the difference between the target function and the input). Based on this concept, ResNet allows features to be directly transmitted through so-called “skip connections”, thereby only requiring the network layers to learn the residuals between inputs and outputs. The output of a residual block is expressed by the following formula:

(3) output=activation(input+F(input))

where F(input) represents the residual function that the network layers need to learn, and activation typically uses the ReLU function. This structure significantly reduces the difficulty of training deep networks, as gradients can propagate directly through the skip connections, thus avoiding the problem of gradient vanishing in multi-layer networks [33].

#### 2.1.3. GoogLeNet

Proposed by Google researchers in 2014 [34], GoogLeNet introduced the Inception module, which significantly enhanced the network’s performance in image recognition tasks. The core idea of the Inception module is to use convolutional kernels of different sizes and pooling operations in parallel within the same layer, enabling the capture of features at various scales within an image. This design allows for the simultaneous processing of local details and broader contextual information, allowing the network to excel across different receptive fields [35]. Typically, an inception module includes 1×1, 3×3, and 5×5 convolutional operations and 3×3 max pooling, with the output being the channel-wise concatenation of these parallel operations. The computation formula for this parallel structure is represented as:

(4) output=parallel(conv1×1,conv3×3,conv5×5,pooling)

Each operation has its independent parameters, enabling the Inception module to adaptively learn the most effective feature combinations. In real-time radish disease detection systems, the ability to effectively handle disease spots of varying sizes and shapes is crucial. The Inception module of GoogLeNet provides excellent technical support for this. For instance, small-sized convolutional kernels can capture fine details of images, while larger kernels help the model understand broader areas of the image, which is vital for identifying and classifying different types of diseases [36]. Additionally, GoogLeNet achieves a balance between network depth and computational efficiency. Although it forms a deep network through the stacking of multiple Inception modules, its design ensures efficient use of computational resources [37]. Each Inception module reduces dimensionality through 1×1 convolutional kernels, not only reducing the number of parameters, but also lowering computational costs, enabling the model to operate efficiently even in resource-limited environments.

### 2.2. Transformer

#### 2.2.1. Transformer in NLP

Since its introduction by Vaswani et al. in 2017 [38], the Transformer model has become a revolutionary architecture for processing sequential data, particularly in the field of Natural Language Processing (NLP) [39,40]. The core innovation lies in the use of the self-attention mechanism, which captures direct dependencies between any two positions within a sequence, significantly enhancing the capacity and efficiency of handling long sequence data. The initial design of the Transformer was intended to address the inefficiencies and gradient vanishing/explosion issues associated with traditional Recurrent Neural Networks (RNN) [41] and Long Short-Term Memory networks (LSTM) [42]. The central mechanism—self-attention—calculates the weight relationships between elements within a sequence, allowing the model to directly access any other part of the sequence at any point in time. The computation formula for the self-attention mechanism is as follows:

(5) $$\mathrm{Attention}\left(Q,K,V\right)=\mathrm{softmax}\left(\frac{QK^{T}}{\sqrt{d_{k}}}\right)V$$

where *Q* (Query), *K* (Key), and *V* (Value) are matrices obtained through different linear transformations of the input data, and $d_{k}$ is the dimension of the key vector, aiding in the stabilization of gradients. Through this mechanism, the output of each element is a weighted sum of all elements in the input sequence, with weights determined by the relative relationships between elements. In the NLP domain, the Transformer model has successfully tackled a variety of complex tasks, including machine translation, text summarization, and sentiment analysis [43,44]. Furthermore, architectures based on the Transformer, such as BERT [45] and GPT [46], have further improved the generalization capabilities and performance on specific tasks through strategies of pre-training and fine-tuning.

#### 2.2.2. Transformer in CV

With the advancement of DL technology, the Transformer model has successfully extended from the domain of natural language processing to computer vision. Particularly, variants like the ViT [47] and Swin Transformer [48] have not only demonstrated the powerful capabilities of the Transformer architecture in visual tasks, but also provided new technological directions for real-time image processing systems, such as radish disease detection systems. The ViT, proposed by Dosovitskiy et al. in 2020, marked the first successful application of the Transformer architecture purely to the visual domain. The core concept of ViT is to segment the image into multiple fixed-size pieces (referred to as patches), which are treated as elements in a sequence, analogous to words in NLP [49]. Each image patch is first flattened and transformed into so-called “patch embeddings” through a linear transformation. Subsequently, these embedding vectors are fed into a standard Transformer model, which processes these sequence data using the self-attention mechanism. The process of ViT can be summarized by the following formula:

(6) $$\mathrm{Patch}\;\mathrm{Embeddings}=\mathrm{Flatten}\left(\mathrm{Image}\;\mathrm{Patch}\right)W_{E}$$

where $W_{E}$ is a learnable matrix that transforms the input image patch into an embedding vector. Within the Transformer, the embedding vectors undergo multiple layers of self-attention and feed-forward networks, ultimately producing outputs for classification or other visual tasks. ViT has exhibited outstanding performance in visual tasks like image classification, demonstrating the potential of the Transformer structure in processing global information within images. For radish disease detection systems, ViT provides a novel perspective by enhancing the understanding and recognition of disease features through global context. To better adapt to complex visual tasks, Liu et al. proposed Swin Transformer, a hierarchical Transformer that enhances model efficiency and flexibility by limiting self-attention to local windows while allowing inter-window connections [48]. The key to Swin Transformer is its window partition strategy, which dynamically adjusts window sizes to adapt to different areas of the image, thus capturing image features more finely. The self-attention computation within the windows can be represented by:

(7) $$\mathrm{Swin}\;\mathrm{Attention}\left(Q,K,V\right)=\mathrm{softmax}\left(\frac{QK^{T}}{\sqrt{d_{k}}}\right)V$$

This design enables Swin Transformer to be highly efficient in processing images, making it particularly suitable for real-time detection systems that require quick responses [50]. In radish disease detection, Swin Transformer can effectively process local feature maps extracted from CNNs, enhancing the model’s precision in disease feature recognition and processing speed through a tiered attention mechanism.

## 3. Materials and Method

### 3.1. Materials

#### 3.1.1. Dataset Collection

**Figure 1:**
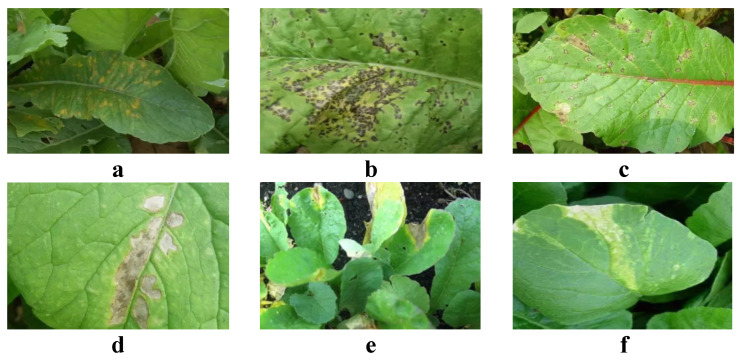
Dataset samples. (**a**) is downy mildew, (**b**) is black spot, (**c**) is anthracnose, (**d**) is bacterial black spot, (**e**) is black rot, (**f**) is viral disease.

In this study, image data of radish diseases such as downy mildew, black spot, anthracnose, bacterial black spot, black rot, and viral diseases were collected, with each disease type amounting to between 1300 and 2000 images, as shown in Figure 1.

The dataset was primarily sourced from publicly available data on the internet and field collections in the Bayannur region of Inner Mongolia Autonomous Region, China. These methods provided a diverse range of radish disease images, which are crucial for building and validating the disease detection model. Data obtained from the internet mainly included annotated images of various disease types, verified by professionals to ensure accuracy and representativeness. Additionally, field collections were conducted in multiple farms in Bayannur, supported and assisted by the local agricultural technology departments. Images from field collections help the model adapt better to variations and diversity in real-world applications, reflecting the actual manifestations of diseases under different climates, soil conditions, and cultivation practices. The number of images collected for each disease is shown in Table 1.

For each disease type, detailed feature descriptions and categorizations were conducted to ensure the dataset’s high quality and applicability. Downy mildew typically presents as white, frost-like powder on leaves, which gradually wither and die as the disease progresses, especially common in humid environments. Black spot is characterized by black, circular, or irregular spots on leaves and roots that often expand over time, leading to tissue necrosis. Anthracnose primarily affects leaves and roots, with leaves displaying watery, dark-brown spots that can lead to the death of the entire leaf. Moreover, bacterial black spot forms dark brown to black, water-soaked spots on leaves, often surrounded by a yellow halo, spreading rapidly throughout the plant. Black rot is a lethal disease affecting the roots, causing the tissue to blacken and soften, ultimately resulting in plant death. Initially, viral disease infections are hard to detect, but as the disease progresses, symptoms such as mottling, curling, and deformation appear on the leaves, severely affecting the growth and yield of radish.

During image collection, high-resolution camera equipment (Canon Mark 3D and iPhone 15 ProMax) was used, ensuring photographs were taken under varying weather conditions and times of the day to capture the disease manifestations under different environmental conditions. All images were preprocessed post-collection, including denoising and adjustments to brightness and contrast, to enhance image quality and the detectability of disease features. Furthermore, to enhance the model’s generalization ability, image augmentation techniques such as random cropping, rotation, and flipping were employed, aiding the model in learning features observed from different angles.

#### 3.1.2. Dataset Preprocessing

**Figure 2:**
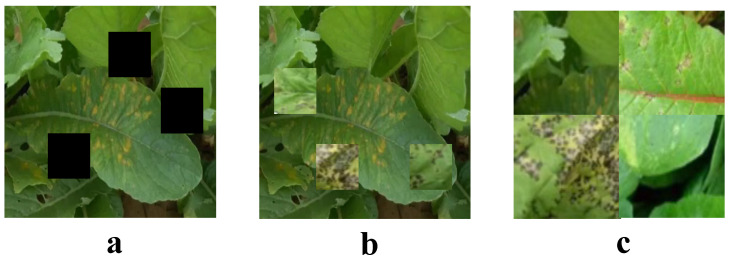
Dataset augmentation. (**a**) is CutOut, (**b**) is CutMix, (**c**) is Mosaic.

In the development process of the real-time radish disease detection system, data augmentation is identified as a crucial step to enhance the model’s generalization capabilities. To effectively adapt to variations in lighting, weather, and background conditions, several data augmentation techniques are employed, including CutOut, CutMix, and Mosaic, as shown in Figure 2. These techniques introduce artificial perturbations into the training data, thereby increasing the diversity and complexity of the training samples, which in turn enhances the model’s ability to recognize unseen types of diseases.

CutOut is a straightforward, yet effective data augmentation method. Its central idea involves randomly selecting a region in the training images and setting its pixel values to zero, thereby simulating the presence of an occlusion. This method forces the model to learn from the remaining parts of the image, enhancing its reliance on local features and increasing its robustness to occlusions in disease recognition. The mathematical expression for CutOut is given by:

(8) $$I^{\prime }\left(x,y\right)=\left\{\begin{array}{cc} 0 & \mathrm{if}\;(x,y)\in \mathrm{mask}\\ I(x,y) & \mathrm{otherwise} \end{array}\right.$$

where I(x,y) represents the pixel value at location (x,y) in the original image, and mask is the randomly generated occlusion area. This approach allows the creation of training challenges without altering the image category labels, thus enhancing the model’s generalization capabilities.

CutMix is a more complex data augmentation technique, which not only introduces occlusions into an image, but also fills the occluded part with a corresponding region from another image. This operation not only increases the diversity of the images, but also enables the model to learn how to blend semantic information between images, which is particularly important for disease classification. The operation of CutMix can be expressed by the following formula:

(9) $$I^{\prime }\left(x,y\right)=\left\{\begin{array}{cc} I_{2}\left(x,y\right) & \mathrm{if}\;(x,y)\in \mathrm{patch}\\ I_{1}\left(x,y\right) & \mathrm{otherwise} \end{array}\right.$$

where $I_{1}$ and $I_{2}$ are the two different images selected for mixing, and patch is the region cut from $I_{2}$. Through this mixture, the model is not only tasked with identifying diseases in the image, but also learning to disregard irrelevant background information, thus enabling more accurate disease detection in complex environments.

Mosaic is a data augmentation method that combines four different images into one. By blending parts of multiple images into a new image, this method forces the model to learn multiple disease features within a single image. The operation of Mosaic can be mathematically represented as follows:

(10) $$I^{\prime }\left(x,y\right)=I_{\mathrm{idx}}\left(x\;\mathrm{mod}\;w_{\mathrm{sub}},y\;\mathrm{mod}\;h_{\mathrm{sub}}\right)$$

where $I_{\mathrm{idx}}$ is one of the four selected images, $w_{\mathrm{sub}}$ and $h_{\mathrm{sub}}$ are the widths and heights of the sub-images, and (x,y) are the coordinates in the new image. idx is determined by the values of *x* and *y*, representing one of the four areas. This integration of multiple images allows the model to simultaneously handle various disease conditions within one view, significantly enhancing its capability to recognize and classify multiple diseases.

Through these data augmentation techniques, the real-time radish disease detection system is better equipped to handle various challenges encountered in practical applications, such as occlusions, changes in lighting, and complex backgrounds. These techniques not only improve the model’s generalization ability, but also enhance its accuracy and robustness in different disease recognition tasks, providing a solid technological support for the real-time detection of radish diseases.

### 3.2. Proposed Method

#### 3.2.1. Overall

**Figure 3:**
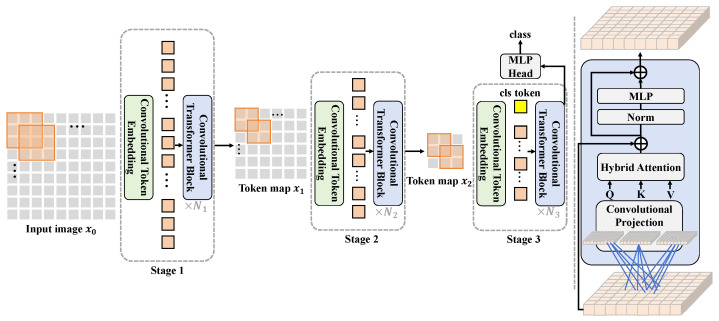
This figure shows the overall architecture of the radish disease detection system based on the hybrid attention mechanism. Starting from the input image, the diagram illustrates the process of progressively extracting and refining features through multiple stages of convolutional transformer blocks and convolutional token embedding, culminating in disease classification via the hybrid attention mechanism and the MLP head. The architecture diagram clearly depicts the detailed process from image input to disease identification and the role of each critical component.

The real-time radish disease detection system proposed in this study employs a hybrid architecture combining CNN and Transformers, aimed at achieving efficient and accurate disease identification. The overall design process revolves around several core modules: the feature extractor, embedding processor, hybrid attention mechanism, and hybrid loss function. These modules are closely linked, forming a coherent processing pipeline to optimize disease detection performance, as illustrated in Figure 3.

Initially, processed image data enter the feature extractor, a module based on deep convolutional neural networks, responsible for extracting basic visual features from the images. During the feature extraction process, through layers of convolutional operations, the network captures various image features ranging from edges and textures to more complex forms and patterns. The mathematical representation of this step is expressed as:

(11) $$x_{1}=f_{\mathrm{CNN}}\left(x_{0}\right)$$

where $x_{0}$ represents the input image data, $f_{\mathrm{CNN}}$ denotes the operations of the convolutional neural network, and $x_{1}$ is the output feature map from the network. Subsequently, these feature maps are fed into the embedding processor. This module’s primary function is to transform the high-dimensional feature vectors into low-dimensional embeddings suitable for processing by the hybrid attention mechanism. This transformation involves a series of fully connected layers that not only reduce dimensionality, but also enhance the model’s expressive capabilities through nonlinear activation functions, such as ReLU. The operation of the embedding processor can be described by the following formula:

(12) $$x_{2}=f_{\mathrm{Embed}}\left(x_{1}\right)$$

where $x_{2}$ is the embedding vector, and $f_{\mathrm{Embed}}$ represents the embedding processing function. The hybrid attention mechanism, central to the system, analyzes the embedding vectors’ and weights’ significant features to highlight information most relevant to disease detection. This mechanism combines spatial and channel attention, dynamically adjusting the focus on features to enhance detection accuracy and efficiency. The computation process of this mechanism is given by:

(13) $$x_{3}=f_{\mathrm{Hybrid}-\mathrm{Attention}}\left(x_{2}\right)$$

where $x_{3}$ is the feature weighted by the attention mechanism, and $f_{\mathrm{Hybrid}-\mathrm{Attention}}$ signifies the computation function of the hybrid attention. Lastly, the hybrid loss function optimizes the overall network parameters by combining cross-entropy loss for classification tasks with Dice loss for addressing sample imbalance, and potentially regularization terms to prevent overfitting, ensuring the model maintains good generalization capabilities. The loss function is formulated as:

(14) $$L=\alpha \cdot L_{\mathrm{cross}-\mathrm{entropy}}+\beta \cdot L_{\mathrm{Dice}}+\lambda \cdot L_{\mathrm{regularization}}$$

where α, β, and λ are weight parameters that regulate the impact of different parts of the loss. Through this comprehensive pipeline from feature extraction to final loss calculation, the system effectively processes input images, accurately identifying and classifying radish diseases. Throughout the process, the close collaboration of the modules and the coherent data flow ensure the system’s high performance and practicality in real-world applications, offering a novel solution in the field of agricultural disease detection.

#### 3.2.2. Feature Extractor

**Figure 4:**
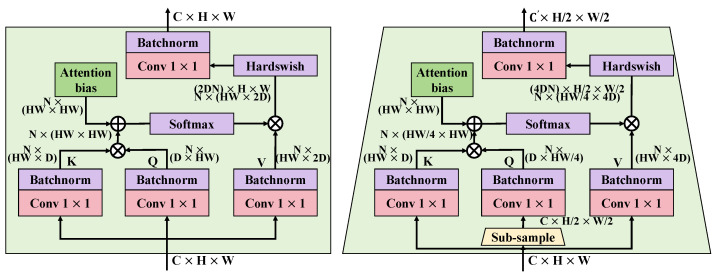
The feature extractor structure diagram. The diagram displays the detailed process of the feature extraction module, including multiple convolutional layers, batch normalization layers, and the interaction of attention mechanisms, gradually transforming the high-dimensional data of the input image into a feature-dense representation, providing precise feature information for subsequent disease recognition and classification.

The design of the feature extractor is crucial for achieving efficient detection of radish diseases in this study. The primary structure of the feature extraction in the system comprises multiple residual blocks, each containing several convolutional layers, batch normalization layers, and ReLU activation functions, as depicted in Figure 4.

1. Input layer: Initially, raw image data are processed through a 7×7 convolutional layer with a stride of 2. This operation is aimed at extracting preliminary image features and reducing dimensionality to decrease computational complexity. Following convolution, max pooling is applied to further reduce the spatial dimensions of the feature map.
2. Residual blocks: The core of the feature extraction network consists of four residual blocks, each structured as follows:
  - Convolutional layer: The first convolutional layer uses 1×1 kernels to compress features, reducing computational complexity for subsequent operations.
  - Convolutional layer: This is followed by a 3×3 convolutional layer designed to extract spatial features.
  - Convolutional layer: The final 1×1 convolutional layer restores the feature dimension and performs residual connections.
  Batch normalization and ReLU activation functions are applied after each convolutional operation to stabilize the training process and enhance non-linear expression capabilities. The output of a specific residual block can be expressed mathematically as:
  (15) $$\mathrm{output}=\mathrm{Activation}\left(\mathrm{BatchNorm}\left(\mathrm{input}+F(\mathrm{input})\right)\right)$$
  where F(input) represents the convolutional operations within the residual block.
3. Output layer: After processing through multiple residual blocks, the feature map undergoes global average pooling to reduce the dimensionality of the final feature vector, preparing a suitable input format for the subsequent embedding processor and hybrid attention mechanism.

Through this structural design, the feature extractor can efficiently extract rich and distinctive features from raw images, providing a solid foundation for accurate identification and classification of radish diseases. Additionally, meticulous settings of network parameters and layer configurations ensure that the model can handle large-scale data while maintaining a rapid response speed, meeting the demands of real-time disease detection.

#### 3.2.3. Embedding Processor

**Figure 5:**
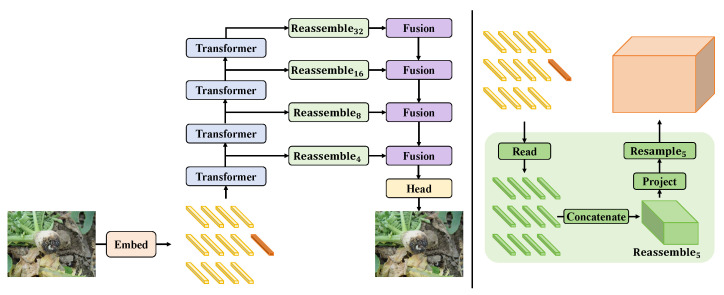
The diagram shows the specific architecture of the embedding processor, including various levels of transformers and fusion processing steps. It details the entire data processing flow from input to output, highlighting the multi-layered fusion and reassembly of information to ensure the richness and adaptability of features during the transformation process.

The embedding processor is a critical module in the real-time radish disease detection system, primarily responsible for transforming the high-dimensional feature maps output by the feature extractor into low-dimensional, dense feature vectors. This transformation facilitates more effective processing by the subsequent hybrid attention mechanism. The design of the embedding processor involves not only dimensionality reduction, but also feature recombination and information fusion to enhance the model’s ability to recognize disease features, as depicted in Figure 5.

The embedding processor employs a multi-layer fully connected network structure, combined with nonlinear activation functions and batch normalization, to effectively convert and embed features. The specific network structure is as follows:

1. Input layer: Initially receives the feature maps from the feature extractor, which typically have high dimensions and depth.
2. Fully connected layer: The feature maps first pass through a fully connected layer for preliminary dimensionality reduction. For instance, if the output from the feature extractor is 1024×7×7 (channels × width × height), the output dimension of this layer might be set to 512. The mathematical representation of the fully connected layer is:
  (16) $$x_{\mathrm{fc}1}=W_{\mathrm{fc}1}x_{\mathrm{input}}+b_{\mathrm{fc}1}$$
  where $W_{\mathrm{fc}1}$ and $b_{\mathrm{fc}1}$ are the weights and bias of the fully connected layer, respectively.
3. Batch normalization and ReLU activation: Following the fully connected layer, batch normalization and ReLU activation functions are applied to enhance the model’s nonlinear processing capabilities and stabilize the training process. This step can be represented as:
  (17) $$x_{\mathrm{relu}}=\mathrm{ReLU}\left(\mathrm{BatchNorm}\left(x_{\mathrm{fc}1}\right)\right)$$
4. Further dimensionality reduction: Subsequently, the data undergo further reduction in dimensionality through a second fully connected layer, potentially reducing to 256 dimensions, to suit further processing requirements. This layer also incorporates batch normalization and ReLU activation:
  (18) $$x_{\mathrm{fc}2}=\mathrm{ReLU}\left(\mathrm{BatchNorm}\left(W_{\mathrm{fc}2}x_{\mathrm{relu}}+b_{\mathrm{fc}2}\right)\right)$$
5. Output layer: Finally, the output layer compresses the data to the required embedding dimension, such as 128 dimensions, which will be directly fed into the hybrid attention mechanism module.

Through the design described above, the embedding processor efficiently transforms high-dimensional feature data into low-dimensional, information-rich embedding vectors. This transformation is crucial for enhancing the efficiency and accuracy of the subsequent attention mechanism. The use of multiple layers for dimensionality reduction and nonlinear activation ensures that features retain rich information during the conversion process and enhances the model’s ability to capture nonlinear relationships, which is particularly important for recognizing complex disease features. The application of batch normalization ensures stability during the training process, accelerates convergence, and the use of ReLU activation prevents the problem of vanishing gradients, ensuring effective training of deep networks.

#### 3.2.4. Hybrid Attention Mechanism

**Figure 6:**
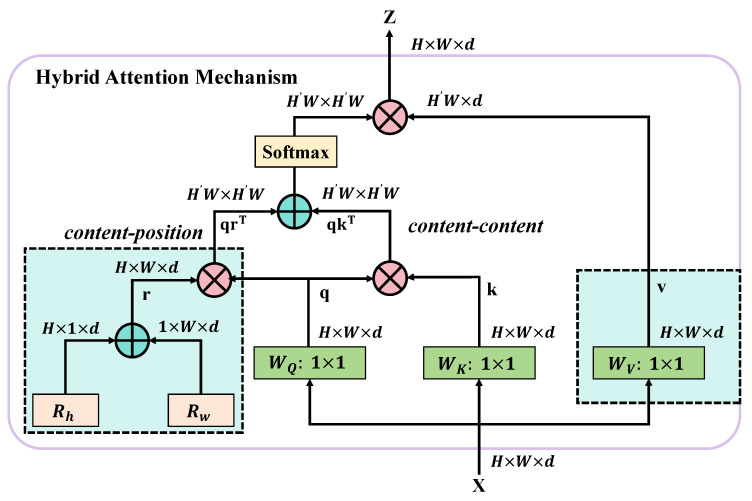
The diagram illustrates the structure of the hybrid attention mechanism. It details the computational process of the hybrid attention mechanism, including the generation of spatial and channel attention and their application on the feature map. This mechanism enhances the model’s ability to recognize disease features in images by integrating weighted features from different dimensions.

The hybrid attention mechanism, a key technology proposed in this study, significantly enhances the performance of the real-time radish disease detection system by combining the advantages of spatial and channel attention. This mechanism provides a more refined feature analysis capability for specific tasks compared to the self-attention mechanism used in traditional Transformer models, especially optimizing both spatial and channel dimensions, as shown in Figure 6.

Traditional Transformer self-attention primarily focuses on relationships between elements in sequence models, updating each element’s state by calculating attention scores across all elements in the sequence. This method excels in sequence processing tasks such as NLP because it efficiently captures long-range dependencies. However, for image data, which have rich spatial information, relying solely on traditional self-attention often fails to fully utilize local spatial features. Therefore, the hybrid attention mechanism proposed in this paper not only finely divides the space, but also considers the dependencies between channels, better adapting to the characteristics of image data. The hybrid attention mechanism initially performs a bidirectional decomposition on the input feature matrix *X*, implemented as follows:

1. Spatial attention mechanism: Weights are assigned to each position in the input feature matrix *X* to emphasize areas more relevant to the target disease features. This process is realized through the following mathematical expression:
  (19) $$S=\mathrm{softmax}\left(\frac{{\mathrm{Conv}}_{1\times 1}\left(X\right)\cdot {\mathrm{Conv}}_{1\times 1}{\left(X\right)}^{T}}{\sqrt{d_{s}}}\right)$$
  Here, ${\mathrm{Conv}}_{1\times 1}$ represents feature transformation through a 1×1 convolutional layer, and $\sqrt{d_{s}}$ is a scaling factor to stabilize the softmax computation.
2. Channel attention mechanism: The importance of each channel is evaluated, prioritizing channels that contain more disease-related information. The computation for channel attention is expressed as:
  (20) $$C=\mathrm{sigmoid}\left(\mathrm{GlobalAvgPool}\left(X\right)W_{c}\right)$$
  where GlobalAvgPool is the global average pooling, and $W_{c}$ represents the weights of a fully connected layer.

Combining these two mechanisms, the final output feature matrix *Z* is obtained through:

(21) Z=(S⊙X)⊗(C⊙X)

where ⊙ represents element-wise multiplication, and ⊗ indicates feature fusion. The design advantage of the hybrid attention mechanism lies in its ability to simultaneously focus on the spatial layout and complex dependencies between channels, which is particularly crucial for image-based tasks. In real-time radish disease detection, this mechanism enables the model to more accurately locate disease areas and identify specific disease types, as different diseases may exhibit varying feature distributions and channel importance visually. Additionally, by precisely controlling the focus of the model’s attention, the response speed and accuracy of the model to subtle disease features are greatly enhanced, crucial for improving the timeliness and reliability of agricultural disease detection. With the application of the hybrid attention mechanism, the system can perform efficient and accurate disease detection in complex agricultural environments, significantly advancing the technological level of radish disease management.

#### 3.2.5. Hybrid Loss Function

In this study, the design of the hybrid loss function is intended to enhance the performance of the radish disease detection model, particularly in addressing sample imbalance and multi-class recognition accuracy. Traditional loss functions, such as cross-entropy loss, are widely used in multi-class problems but often exhibit limited performance with imbalanced data. Consequently, a hybrid loss function combining cross-entropy loss and Dice loss has been proposed to overcome this limitation. The hybrid loss function consists of two parts: cross-entropy loss and Dice loss. Cross-entropy loss, a common loss function in classification problems, measures the discrepancy between the probability distribution predicted by the model and the actual distribution of the labels. Its mathematical expression is:

(22) $$L_{\mathrm{CE}}=-\sum_{i=1}^{C}y_{i}\log\left({\hat{y}}_{i}\right)$$

where *C* represents the total number of classes, $y_{i}$ is the true label for class *i*, and ${\hat{y}}_{i}$ is the probability predicted by the model for class *i*. Dice loss, originating from the medical imaging field, is particularly suited for addressing data category imbalance. It is based on a set similarity measure that effectively evaluates the similarity between two samples. The definition of Dice loss is:

(23) $$L_{\mathrm{Dice}}=1-\frac{2\sum_{i=1}^{C}y_{i}{\hat{y}}_{i}}{\sum_{i=1}^{C}y_{i}^{2}+\sum_{i=1}^{C}{\hat{y}}_{i}^{2}}$$

Here, $y_{i}$ and ${\hat{y}}_{i}$ represent the true and predicted values, respectively; the numerator is twice the intersection size of the true and predicted values, and the denominator is the sum of the squares of each. Combining these two losses, the hybrid loss function can be expressed as:

(24) $$L_{\mathrm{Hybrid}}=\alpha L_{\mathrm{CE}}+\beta L_{\mathrm{Dice}}$$

where α and β are hyperparameters used to balance the contributions of the two losses. While cross-entropy loss effectively optimizes the accuracy of the model’s classifications, it can bias the model towards the majority class in the presence of extremely imbalanced data, overlooking the minority class predictions. Incorporating Dice loss compensates for this deficiency by directly focusing on the overlap between the predicted and true regions of the classes, thereby enhancing the model’s learning towards minority classes. From a mathematical perspective, Dice loss optimizes the ratio between predicted and true values, naturally focusing on a balanced representation of all classes. This optimization of the overlapping area ensures that the model can attempt to find optimal boundaries for all classes, even in cases of severe data imbalance.

In summary, the introduction of the hybrid loss function enables the radish disease detection model to effectively address sample imbalance issues while ensuring the accuracy of classification tasks, enhancing the model’s generalization ability and practicality. By appropriately adjusting the values of α and β, further optimization of model performance can be achieved, ensuring that the model is both efficient and accurate in its disease detection capabilities when deployed in practical settings.

### 3.3. Experimental Setup

#### 3.3.1. Hardware and Software Platform

The core of the experimental platform is constituted by servers equipped with NVIDIA GPUs. The formidable computational power of GPUs enables parallel processing of large-scale data, significantly accelerating the training and validation process of models. Particularly effective for performing extensive matrix operations, GPUs are crucial for the forward and backward propagation processes in DL. Specifically, the chosen GPUs feature high memory bandwidth and numerous CUDA cores, allowing for the rapid processing of complex computations found in convolutional neural networks and Transformer networks.

In addition to hardware configuration, the choice of software environment is a key factor in optimizing experimental efficiency. The Python programming language, supporting a rich library and framework ecosystem, is adopted in the laboratory. The PyTorch framework is selected for DL tasks due to its optimized underlying code that fully utilizes GPU resources, providing efficient data handling and model training capabilities. These experimental platforms not only facilitate effective processing and learning of large-scale agricultural image data, but also promote the research and application of real-time radish disease detection technology by ensuring experimental efficiency.

#### 3.3.2. Hyperparameter Settings

In the construction of the real-time radish disease detection system, appropriate hyperparameter settings are crucial for the training effect and ultimate performance of the model. The choice of hyperparameters not only affects the speed of model learning, but also directly relates to the model’s fit to data and generalization ability. The learning rate, one of the most important hyperparameters in DL, determines the step size for model weight updates. If the learning rate is set too high, the model may exhibit oscillations during training or even diverge, preventing convergence to an optimal solution. Conversely, a learning rate that is too low may slow down the training process or cause the model to become stuck in local minima. In this project, the learning rate is set at 0.001. Additionally, the Adam optimizer is employed for adaptive adjustment of the learning rate. Combining the benefits of AdaGrad and RMSProp optimizers, the Adam optimizer dynamically adjusts the learning rate based on the mean of the squares of the gradients for each parameter and also incorporates an estimation of the first moment of gradients, i.e., momentum. This makes the Adam optimizer exhibit better stability and faster convergence in practical applications. The parameter update formula for the Adam optimizer is given as follows:

(25) $$\theta_{t+1}=\theta_{t}-\frac{\eta }{\sqrt{{\hat{v}}_{t}}+\epsilon }{\hat{m}}_{t}$$

where $\theta_{t}$ is the parameter at time *t*, η is the learning rate, ${\hat{m}}_{t}$ and ${\hat{v}}_{t}$ are estimations of the first (mean) and second (uncentered variance) moments, and ϵ is a small constant added for numerical stability (typically set to 1 × 10^−8^. The batch size directly impacts memory consumption and the frequency of updates during model training. A larger batch size can improve memory utilization and computational efficiency since modern computing platforms, like GPUs, are generally optimized for large-scale matrix operations. However, too large a batch size may reduce the variance in gradient estimates during the training process, thereby increasing the risk of converging to local minima. In this project, the batch size is set to 32, a compromise that ensures efficiency while avoiding excessive smoothing of gradient estimates.

The number of training epochs is another key hyperparameter, determining how many times the entire training set is used to train the model. Too few training epochs may lead to underfitting, where the model fails to learn sufficient features from the data; conversely, too many epochs may result in overfitting, where the model learns the training data too well, leading to decreased generalization ability. The plan is to conduct 100 training epochs to ensure that the model sufficiently learns at various stages without overfitting.

### 3.4. Evaluation Metrics

The evaluation metrics employed in this project include Recall, Precision, Accuracy, Mean Average Precision (mAP), and Frames Per Second (FPS). Recall is an important performance metric, especially in tasks like disease detection, where sensitivity to missed detections is crucial. It measures the proportion of actual positives correctly identified by the model. Precision focuses on the proportion of actual positives among the samples identified by the model as positives. Accuracy, the most intuitive performance metric, indicates the proportion of correct predictions among all predictions made. mAP, an important metric for assessing detection model performance, particularly in object detection tasks, calculates the average of precision over different levels of recall, providing a comprehensive performance evaluation for disease detection systems:

(26) $$\mathrm{mAP}=\int_{0}^{1}p\left(r\right),dr$$

where p(r) is the area under the precision–recall curve. mAP considers not only the accuracy of detections, but also their completeness, serving as a comprehensive indicator of how the model performs across various operational points. FPS, a key metric for assessing real-time processing capabilities, especially in systems requiring quick responses, reflects the time taken by the system to process each frame of the image. A higher FPS indicates that the system can analyze images and respond more quickly, which is crucial for real-time monitoring of disease progression. These comprehensive evaluation metrics enable a thorough analysis and optimization of the radish disease detection system’s performance, ensuring its reliability and effectiveness in actual agricultural production.

## 4. Results and Discussion

### 4.1. Disease Detection Results

In this study, the purpose of the experiment was to evaluate the performance of different disease detection models on radish disease detection tasks, particularly in terms of precision, recall, accuracy, mAP, and FPS. These metrics comprehensively assess the practical effectiveness of each model in disease detection, including their accuracy, miss rate, overall classification effectiveness, and real-time performance. The FPS metric is especially critical for agricultural application scenarios that require rapid response in disease detection systems, thus the experiment not only examined the accuracy of models, but also focused on their performance in real-world applications.

From the experimental results presented in Table 2, it is observed that the DETR model had a precision of 0.81, a recall of 0.78, an accuracy of 0.79, an mAP of 0.80, and an FPS of 20. Although DETR could accurately identify diseases to some extent, its relatively slow detection speed and low FPS may be due to the computational overhead associated with its global dependency calculations in handling large image features using self-attention mechanisms. The YOLOv8 and CenterNet models showed improvements in both accuracy and speed, particularly YOLOv8, which had a precision of 0.83 and an FPS of 26. This enhancement in speed is attributed to YOLOv8’s optimized convolution operations in target detection. CenterNet, with a precision of 0.85 and an FPS of 32, excels by locating targets through keypoint detection rather than traditional bounding box methods, offering more precise target localization and thus higher detection accuracy. TinySegformer displayed higher precision at 0.87 and an FPS of 38, outperforming earlier models with its lightweight structure and refined segmentation capabilities, suitable for practical applications. Compared to the previous models, YOLOv10 showed further improvements in all metrics, particularly in FPS, reaching 44, indicating its excellent real-time processing capability while maintaining high accuracy. The ECOS model performed exceptionally with a precision of 0.91 and an FPS of 50, thanks to its optimized feature extraction and target detection algorithms that effectively balance speed and accuracy. Lastly, the method proposed in this paper exhibited the best performance across all metrics, especially with a precision of 0.93 and an FPS of 57. This superior performance is largely due to the hybrid attention mechanism and heterogeneous loss function designed in this study, enabling the model to simultaneously focus on both local and global image features and effectively handle data imbalances.

Theoretically, the DETR model, utilizing self-attention mechanisms, captures global dependencies effectively but at a high computational cost, impacting its speed. YOLOv8 and YOLOv10, with optimized convolution operations and lightweight design, improve detection speeds while maintaining high accuracy through an efficient feature pyramid structure and multi-scale detection mechanisms. CenterNet’s approach to using keypoint detection reduces reliance on bounding boxes for target localization, offering more accuracy but potentially increasing the risk of missing complex diseases. TinySegformer reduces computational overhead and enhances response speeds through smaller network parameters and an efficient attention mechanism, maintaining sensitivity to image details for complex disease detection tasks. ECOS combines multi-scale feature fusion and optimized attention mechanisms, dynamically adjusting focus on different features, thus achieving well-rounded performance in complex scenarios. The method developed in this study innovates with a hybrid attention mechanism that captures nuanced local features and broader global context, resolving detection bottlenecks caused by asymmetric feature information mathematically. Additionally, the heterogeneous loss function considers class imbalance challenges during optimization, a novel approach that ensures high detection accuracy across various disease types. Through theoretical analysis, it is evident that the mathematical optimizations and innovative designs in this study enable breakthrough improvements in both accuracy and real-time performance.

### 4.2. Results Analysis

The experimental design of this paper primarily aims to evaluate and demonstrate the performance of the proposed disease detection model in handling various types of radish diseases, including downy mildew, black spot, anthracnose, bacterial black spot, black rot, and viral diseases of radishes. The results of the experiments are presented in Table 3.

From the results, it is observed that the model’s success across various diseases can largely be attributed to the optimization of its mathematical features. The hybrid attention mechanism utilized by the model acts on both spatial and channel dimensions, enhancing sensitivity and recognition capabilities towards disease characteristics through finely tuned learned feature weights. Moreover, the model structure’s hybrid loss function further optimizes the learning process, particularly under sample imbalance conditions, by combining Dice loss and cross-entropy loss. This enhances the model’s learning capabilities for minority class samples, thereby improving recall and accuracy. Such mathematical designs ensure that the model not only excels in recognition accuracy, but also meets the real-time processing speeds required for agricultural field applications. In conclusion, the model, with its mathematical modeling optimizations and innovations, performs excellently in detecting various types of radish diseases, especially those with complex symptom manifestations. By further adjusting and optimizing model parameters, it is expected to provide even greater efficacy in future practical applications, offering robust technical support for agricultural disease management.

### 4.3. Ablation Study on Different Attention Mechanisms

This experiment aims to compare the effects of different attention mechanisms on the performance of radish disease detection models, specifically analyzing the efficacy of each attention mechanism to validate the advantages of the hybrid attention mechanism. The primary purpose of the experimental design is to explore the performance differences of various types of attention mechanisms, such as standard self-attention, Convolutional Block Attention Module (CBAM), and Hybrid Attention in disease detection tasks.

From the results in Table 4, it is observed that the standard self-attention mechanism performs relatively lower, with a precision of 0.76, recall of 0.72, accuracy of 0.74, mAP of 0.75, and FPS of 43. Although this traditional self-attention mechanism can capture global dependencies, it may underperform in specific image recognition tasks due to a lack of effective capture of local features. Mathematically, standard self-attention updates features by calculating the weight distribution across all element pairs, which, in visual tasks, might be overly generalized, failing to effectively distinguish critical local information within the image. In contrast, the CBAM shows improvement across all metrics, with a precision of 0.84, recall of 0.80, accuracy of 0.83, mAP of 0.83, and FPS of 51. CBAM optimizes feature expression by combining spatial and channel attentions, allowing the model to better focus on important areas of the image. CBAM’s design philosophy sequentially focuses on channel and spatial features, enhancing the specificity and effectiveness of attention through a two-step refinement process. Mathematically, this method initially captures the statistical features between channels through global average pooling and max pooling, then transforms and activates these features, enhancing the model’s ability to differentiate the importance of various channels. Spatial attention further focuses on specific spatial locations through convolution operations, strengthening the expression of local features. Lastly, the hybrid attention mechanism exhibits the best performance, with a precision of 0.93, recall of 0.89, accuracy of 0.91, mAP of 0.90, and FPS of 57. The superiority of the hybrid attention mechanism lies in its ability to simultaneously process spatial and channel information, providing a more comprehensive and detailed method of feature analysis. Mathematically, hybrid attention not only considers the interactions between elements, but also specifically enhances focus on critical parts of the image by dynamically adjusting the weights of each channel and spatial position, precisely controlling the focus of information flow, thus significantly enhancing detection accuracy and efficiency.

### 4.4. Ablation Study on Different Loss Functions

This experiment aims to assess the impact of different loss functions on the performance of radish disease detection models, as shown in Table 5. The primary objective of the experimental design is to compare the commonly used cross-entropy loss, focal loss, and the proposed hybrid loss introduced in the study to determine their suitability and advantages in radish disease detection tasks, particularly under conditions of sample imbalance or category diversity.

From the experimental results, it is observed that the model using cross-entropy loss exhibits moderate performance with a precision of 0.74, recall of 0.70, accuracy of 0.72, mAP of 0.72, and an FPS of 48. Cross-entropy loss, being the most common loss function for classification problems, calculates the loss by comparing the probability distribution of the model outputs with the actual labels, mathematically represented as the negative log-likelihood. This loss function is suitable for cases with balanced categories, but its effectiveness may be limited when dealing with imbalanced data or multi-category datasets, as it does not adequately consider the imbalance between categories, leading the model to favor the majority class. In contrast, the model utilizing focal loss shows improved performance across all metrics, with a precision of 0.82, recall of 0.79, accuracy of 0.81, mAP of 0.80, and an FPS of 53. Focal loss is an enhancement to cross-entropy loss designed to address class imbalance issues, particularly in cases where one class significantly outnumbers others. Focal loss modifies the cross-entropy formula by adding a modulating factor ${\left(1-p_{t}\right)}^{\gamma }$, where $p_{t}$ is the probability of correct classification for each class by the model, and γ is a tuning parameter. This design reduces the loss contribution from easy-to-classify samples, focusing the model’s attention on more challenging examples, thereby improving the recall of minority classes and overall model performance. Finally, the hybrid loss exhibits the best performance, achieving a precision of 0.93, recall of 0.89, accuracy of 0.91, mAP of 0.90, and an FPS of 57. Hybrid loss combines the advantages of cross-entropy and Dice Losses, where Dice Loss is particularly suited for handling class imbalance issues, as it measures the similarity between classes, directly optimizing the differentiation between categories. The design of hybrid loss allows the model to simultaneously optimize classification accuracy and inter-class segmentation effectiveness, balancing these two components within the loss function to effectively recognize and classify complex disease features.

### 4.5. Limits and Future Works

In this study, we successfully developed a radish disease detection system based on a hybrid attention mechanism, which exhibited superior performance across several key performance indicators. However, despite significant achievements, there remain some limitations and shortcomings that need to be addressed and optimized in future work. Firstly, although the proposed hybrid attention mechanism has demonstrated good performance in experiments, it has a high computational complexity, especially when processing large-scale datasets. The hybrid attention mechanism requires calculating attention distributions across both spatial and channel dimensions, which increases the model’s parameter count and computational burden. In resource-limited application scenarios, such as mobile devices or edge computing platforms, this may lead to slower processing speeds, affecting the system’s real-time response capabilities. Future research should explore more effective algorithms or optimization strategies to reduce computing resource consumption while maintaining model performance. This aspect of the research can refer to [19], which proposes an efficient model architecture that reduces resource consumption and processing time for tomato disease detection against complex backgrounds.

Although this study used a hybrid loss function to address the issue of class imbalance, achieving high precision and recall rates, the model’s performance still needs improvement under extremely imbalanced data conditions. Particularly for some rare or difficult-to-identify disease categories, the model may not fully meet practical application requirements. Future work could consider introducing more advanced data augmentation techniques, such as using Generative Adversarial Networks (GANs), to synthesize training samples for rare diseases, or developing more refined sample weighting strategies to further enhance the model’s ability to recognize minority categories. Research in this area can refer to [15], which discusses the potential and practical applications of using artificial intelligence technology to address similar issues in agriculture.

Moreover, although the current study was specifically designed and optimized for radish disease detection, its universality and performance in detecting diseases in other crops still need further validation. Future research could extend the current model’s application scope to explore its effectiveness in other crop disease detections, and make appropriate adjustments and optimizations according to the characteristics of different crops. In this regard, “Role of Internet of Things and Deep Learning Techniques in Plant Disease Detection and Classification: A Focused Review” [20] provides a framework and examples of integrating IoT and deep learning technologies, which are beneficial for expanding the model’s application scenarios.

Finally, more in-depth research is needed on how to translate these technologies into actual economic benefits in future work. This includes developing cost–benefit analysis models to assess the impact of technology implementation on agricultural production costs, crop yield, and farmers’ income. Additionally, the social and environmental impacts of technology dissemination should be considered, including the positive effects of reducing pesticide use on the environment and the potential impact of technology application on the agricultural labor market [21]. In summary, although this research has achieved certain results, there is still considerable room for improvement in theoretical depth and application breadth. By improving existing models and algorithms, as well as continuously optimizing and expanding the dataset, future research is expected to achieve more accurate, efficient, and intelligent radish disease detection systems, better serving the needs of modern agriculture.

## 5. Conclusions

In this paper, we developed a radish disease detection system based on a hybrid attention mechanism, aimed at enhancing the accuracy and efficiency of agricultural disease detection. The main innovation and contribution of this paper lie in the introduction of the hybrid attention mechanism, which combines the advantages of spatial and channel attentions, optimizing the limitations of traditional attention mechanisms in processing image data. While traditional self-attention mechanisms can handle long-range dependencies in sequential data, visual tasks, particularly in disease image detection, often require more detailed local feature analysis capabilities. The hybrid attention mechanism, by comprehensively analyzing the spatial and channel features of images, enhances the model’s sensitivity to disease characteristics and accuracy in classification. The experimental results demonstrate that the model proposed in this paper excels across multiple key performance metrics. Compared with traditional self-attention mechanisms, CBAM, and other advanced attention models, our model shows significant improvements in precision, recall, and accuracy. Specifically, the proposed method achieved a precision of 93%, recall of 89%, and an accuracy of 91%, with an mAP of 90%, significantly surpassing other comparative models. Additionally, the FPS of our model reached 57, indicating that while maintaining high accuracy, the model also possesses good real-time processing capabilities. Furthermore, this study validated the effectiveness of the hybrid loss function in addressing class imbalance issues through different loss function ablation experiments. Compared to traditional cross-entropy loss and focal loss, the hybrid loss function not only improved the model’s precision in detecting minority class diseases, but also enhanced the overall detection performance. This was clearly evidenced in the experimental results, where the model using the hybrid loss function outperformed those using just cross-entropy loss or focal loss on all performance metrics. Through this research, we not only proposed a novel method for disease detection, but also effectively improved and expanded existing disease detection technologies. Future work will continue to explore more efficient algorithms and model structures to further enhance the accuracy and real-time capabilities of disease detection. Additionally, we plan to expand the dataset to include more types of diseases and more complex application scenarios to comprehensively verify and enhance the model’s generalizability and practicality. In summary, the findings of this paper provide new perspectives and methods for the field of agricultural disease detection, holding significant theoretical and practical significance for advancing smart agriculture.

## Figures and Tables

**Table 1:** Quantity of data for different diseases.

Disease	Quantity
Downy mildew	1398
Black spot	1701
Anthracnose	1927
Bacterial black spot	1493
Black rot	1566
Viral disease	1882

**Table 2:** Disease detection results.

Model	Precision	Recall	Accuracy	mAP	FPS
DETR [51]	0.81	0.78	0.79	0.80	20
YOLOv8 [52]	0.83	0.80	0.81	0.82	26
CenterNet [53]	0.85	0.82	0.83	0.83	32
TinySegformer [54]	0.87	0.84	0.85	0.85	38
YOLOv10 [55]	0.89	0.85	0.87	0.87	44
ECOS [56]	0.91	0.87	0.89	0.88	50
Proposed Method	0.93	0.89	0.91	0.90	57

**Table 3:** Results analysis.

Disease	Precision	Recall	Accuracy	mAP
Downy mildew	0.89	0.85	0.87	0.88
Black spot	0.90	0.87	0.88	0.88
Anthracnose	0.92	0.88	0.90	0.91
Bacterial black spot	0.93	0.88	0.91	0.90
Black rot	0.95	0.90	0.93	0.92
Viral disease	0.96	0.92	0.94	0.93

**Table 4:** Ablation study on different attention mechanisms.

Attention Mechanism	Precision	Recall	Accuracy	mAP	FPS
Standard Self-Attention [57]	0.76	0.72	0.74	0.75	43
CBAM [58]	0.84	0.80	0.83	0.83	51
Hybrid Attention	0.93	0.89	0.91	0.90	57

**Table 5:** Ablation study on different loss functions.

Loss Function	Precision	Recall	Accuracy	mAP	FPS
Cross-Entropy Loss [59]	0.74	0.70	0.72	0.72	48
Focal Loss [60]	0.82	0.79	0.81	0.80	53
Hybrid Loss	0.93	0.89	0.91	0.90	57

## Data Availability

The data presented in this study are available on request from the corresponding author.
